# Decreased YB‐1 expression denervates brown adipose tissue and contributes to age‐related metabolic dysfunction

**DOI:** 10.1111/cpr.13520

**Published:** 2023-06-15

**Authors:** Ruoyu Zhou, Yan Huang, Xu Feng, Rui Zhou, Liwen Wang, Genqing Xie, Yuan Xiao, Haiyan Zhou

**Affiliations:** ^1^ Department of Endocrinology, Endocrinology Research Center Xiangya Hospital of Central South University Changsha China; ^2^ Department of Endocrinology The First People's Hospital of Xiangtan city Xiangtan China; ^3^ National Clinical Research Center for Geriatric Disorders Xiangya Hospital Changsha China

## Abstract

Thermogenesis in brown adipose tissue (BAT) declines with aging, however, the underlying mechanism remains unclear. Here, we show that the expression of Y‐box binding protein 1 (YB‐1), a critical DNA/RNA binding protein, decreased in the BAT of aged mice due to the reduction of microbial metabolite butyrate. Genetic ablation of YB‐1 in the BAT accelerated diet‐induced obesity and BAT thermogenic dysfunction. In contrast, overexpression of YB‐1 in the BAT of aged mice was sufficient to promote BAT thermogenesis, thus alleviating diet‐induced obesity and insulin resistance. Interestingly, YB‐1 had no direct effect on adipose UCP1 expression. Instead, YB‐1 promoted axon guidance of BAT via regulating the expression of Slit2, thus potentiating sympathetic innervation and thermogenesis. Moreover, we have identified that a natural compound Sciadopitysin, which promotes YB‐1 protein stability and nuclear translocation, alleviated BAT aging and metabolic disorders. Together, we reveal a novel fat‐sympathetic nerve unit in regulating BAT senescence and provide a promising strategy against age‐related metabolic disorders.

## Graphical abstract

In this study, we found that dysregulated butyrate levels in aging mice decreased YB‐1 expression in the brown adipose tissue (BAT), which aggravates aging‐related BAT dysfunction. Mechanistically, YB‐1 promotes BAT function by facilitating sympathetic innervation via producing Slit2. Importantly, we have identified a natural compound, sciadopitysin, could maintain the expression of YB‐1 in the BAT, thus alleviating aging‐related BAT dysfunction.

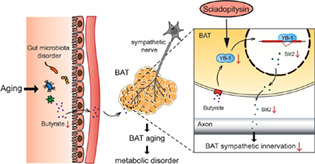

## 1 INTRODUCTION

Brown adipose tissue (BAT) is responsible for non‐shivering thermogenesis, which is important for maintaining metabolic health. With increasing of age, BAT gradually becomes senescent characterized by increased adiposity, inflammatory immune cell infiltration, and declined thermogenesis function, leading to the onset of obesity and age‐related metabolic disorders.^1^ It is reported that deficiency of impaired mitochondrial function, decreased sympathetic nerve distribution, declined adipose stem cell function, as well as mussy endocrine signalling are associated with BAT aging, but the detailed mechanisms has not been fully elucidated.

Short chain fatty acids (SCFAs) are produced from the breakdown of indigestible dietary carbohydrates by gut microbiota and are the main energy source for colonic epithelial cells.^2^ SCFAs mainly include acetic acid, propionic acid and butyric acid. Among them, butyrate has been shown to maintain the integrity of the intestinal epithelial barrier function and the stability of the inflammatory environment.^3^, ^4^ Studies have shown that butyrate levels are significantly reduced in the gut of inflammatory disease, metabolic disease and aging individuals.^4^, ^5^ Butyrate can bind and activate G protein‐coupled receptors (GPR) GPR41, GPR43 and GPR109A, which are widely present in intestinal epithelial cells and extraintestinal tissues and organs. Among them, GPR43 is more highly expressed in adipose tissue, and its expression is related to high‐fat diet and SCFA feeding.^6^, ^7^

Y box‐binding protein 1 (YB‐1), a critical of DNA/RNA binding protein, is involved in the regulation of DNA replication and repair, mRNA transcription, splicing, stability and translation.^8^, ^9^ YB‐1 expression was suppressed in six tissues of aged rats including BAT, while calorie restriction, which attenuated aging‐related changes, restored its expression.^10^ We have previously reported that YB‐1 protein is down‐regulated in hypothalamic neural stem cells of aged mice and maintain YB‐1 expression protects against aging‐associated physiological decline.^11^ A number of studies have found a negative association of YB‐1 protein with thermogenic function.^12^, ^13^ However, these studies focused on the role of YB‐1 in preadipocytes or mesenchymal stem cells during which YB‐1 deficiency mainly affect thermogenic function via decreasing adipocyte differentiation capacity.^12^ But the role and the key targets of YB‐1 in mature adipocytes at post‐transcriptional level during BAT aging are largely unknown.

In this study, we found that the decline of YB‐1 in BAT of aging mice ablated sympathetic innervation by reducing Slit2 expression, leading to BAT aging and exacerbated aging‐related obesity and other metabolic syndromes. In addition, we found that the natural compound, sciadopitysin can promote the expression of YB‐1 and improve the function of aging BAT.

## 2 MATERIALS AND METHODS

### 2.1 Mice and animal care

YB‐1^flox/flox^ mice and *adiponectin*‐cre mice were purchased from the Cyagen Biosciences. Wild‐type mice aged were purchased from Hunan Laboratory Animal Company (SJA, Hunan, China). Mice were fed with normal chow diet (ND) or high fat diet (HFD) containing 60% kcal from fat, 20% kcal from carbohydrate, and 20% kcal from protein (D12492, Research Diets) for 12 weeks at 6 weeks of age. All animals were maintained on 12 h light:12 h darkness cycles (lights on at 06:00 h) and allowed access to diet and water ad libitum. All animal protocols for this study were reviewed and approved by the Animal Care and Use Committees of the Laboratory Animal Research Center at Xiangya Medical School of Central South University.

### 2.2 Mice treatment

Butyrate sodium (Sigma), which were dissolved in sterile saline (0.9% w/v) and filtered with 0.22 μm filter membrane. Mice were intraperitoneally injected with sodium butyrate (40 μg/kg) 3 time a week for 2 weeks and YB‐1 expression was measured in the BAT. Slit2 was intravenously injected in to mice 3 time a week for 2 weeks and cold stimulation experiments were performed. Sciadopitysin was prepared into 1 ug/ul solution and gavaged according to the weight of mice 10 mg/kg/d (*n* = 5). The control group was given the same normal saline (*n* = 5). Gavage was conducted 3 times a week for 10 weeks. The weight of mice was monitored weekly.

### 2.3 Microbial transplantation

Elimination of intestinal flora in mice: mix ampicillin (1 mg/mL), metronidazole (1 mg/mL), neomycin (1 mg/mL) and vancomycin (0.5 mg/mL) in drinking water. Change the water every 3 days and feed for 2 weeks.

Faecal microbiome transplantation (FMT): 6 mice of 8 weeks age in each group of recipient mice (yFMT and oFMT). Donor young mice were 8 weeks old, and aged mice were 15 months old. Take the faeces of young mice and aged mice was gathered and placed in sterile PBS. Then crushed them and centrifuged for 20 times and took the supernatant. Gavage the mice with a dose of 30 ul suspension per mice. Gavage continued for 3 weeks, 3 times a week.

### 2.4 Cold stress

The mice were placed in a separate cage with a small amount of padding with food and water. First measure the core temperature of the mice with an anal thermometer at room temperature, and then place the mice in the cold storage to keep the temperature at 6–8°C. Observe the state of mice every hour and measure the core body of mice. After 8 h, the mice were decapitated and killed.

### 2.5 Glucose tolerance test and insulin tolerance test

The mice were pre stimulated for 1 week to make them adapt to the shock of blood glucose measurement in advance. Start fasting overnight for 12–16 h the night before GTT. The fasting blood glucose of mice was measured in the morning of the next day, and then 20% glucose was injected intraperitoneally according to 10 UL/g body weight. The blood glucose of mice was measured at 15, 30, 60, 90 and 120 min after intraperitoneal injection. ITT would be conducted 1 day after GTT. Mice were fasted for 4–6 h. The mice were injected intraperitoneally with insulin glargine of 0.75 units/kg after fasting blood glucose was measured at 15, 30, 60, 90 and 120 min after insulin injection.

### 2.6 AAV‐YB‐1 virus BAT in situ injection

YB‐1 overexpression adeno‐associated virus was purchased from Hanheng Biology and injected into BAT of mice of 12‐month‐old (*n* = 8) in situ at the concentration of 1. AT. The control group (*n* = 8) was injected with empty vector. The procedure of in situ injection is as follows. The mice were anaesthetised with 0.15 mL/20 g of 1% Pentobarbital. The anaesthetised mice were placed on the workbench, and a 2 cm surgical incision was cut on the back of the mice after shaving and disinfection. Carefully separate the fascia and white adipose tissue around the BAT under the skin, and inject 3 ~ 4 points of bat on both sides with a micro sampler. After completion, the skin at the edge of the surgical incision was disinfected with compound iodine, sutured, and then disinfected again.

### 2.7 ELISA

The detection of Slit2 was conducted using a mouse Slit2 ELISA Kit (Colorimetric) according to the manufacture's guidance.

### 2.8 Cell culture

C3H10T1/2 preadipocytes were cultured in DMEM (Gibco) supplemented with 10% foetal bovine serum (Gibco), 1% penicillin/streptomycin solution (Solarbio) at 37°C in a humidified incubator with 5% CO_2_. For adipogenic differentiation, confluent C3H10T1/2 preadipocytes were incubated with complete medium supplemented with 0.5 mM 3‐isobutyl‐1‐methylxanthine (Sigma), 1 μM dexamethasone (Sigma), 850 nM insulin (Sigma), 1 nM triiodothyronine (Sigma), 125 nM indomethacin (Sigma) and 1 μM rosiglitazone (MedChemExpress) for 2 days. Then, cells were changed to the complete medium supplemented with 1 nM T3, 850 nM insulin and 1 μM rosiglitazone for another 2 days. Differentiated adipocytes at day 4 were maintained in complete medium until used for experiments. Lipid droplets in mature adipocytes were detected by Oil Red O staining. For stimulation of adipocytes with butyrate sodium, cells were treated with 0.5 mM butyrate sodium, a concentration that is sufficient to up‐regulate the expression of thermogenesis‐related genes in BAT stromal vascular fraction (SVF) cells,^14^ for 24 h.

PC12 cells were maintained in RPMI‐1640 (Gibco) supplemented with 10% FBS (Gibco) and 1% penicillin/streptomycin solution (Solarbio). To induce PC12 cell differentiation, cells were treated with 50 ng/mL NGF (Peprotech) for 5 days in low‐serum medium (2% FBS) and the medium was replaced every 2 days. After 5‐day induction, cells were fixed with 4% paraformaldehyde, and proceeded to perform immunofluorescence staining to determine neurite outgrowth of PC12 cells. Images were representative of three independent experiments. Neurite lengths were measured from a total of 4 ~ 7 wells per group by Image J.

### 2.9 Western blot

Proteins from tissues and cells were obtained using RIPA buffer (Pierce, Rockford, IL) supplemented with a protease inhibitor cocktail (Roche, Mannheim, Germany), and quantitated for concentrations with Bradford method (Pierce, #23238). The proteins lysates were heated at 95°C for 5 min in sample buffer containing 2% SDS and 1% 2‐mercaptoethanol, separated on 10% or 12% SDS–polyacrylamide gels, and transferred to PVDF membranes using a wet transfer apparatus (Bio‐Rad). The membranes were blocked with 5% BSA for 1 h and then incubated with antibodies against YB‐1, phosphorylated YB‐1, GAPDH, P21, PGC1α, PRDM16, PPARγ, FABP4, β‐ACTIN, and UCP1 (All above antibodies were purchased from Cell signalling except UCP1 antibody was purchased from Abcam) overnight followed by a horseradish peroxidase‐conjugated anti‐rabbit or anti‐mouse IgG. Signals were detected with ChemiDocTM XRS+ (Bio‐Rad) with Image LabTM Software.

### 2.10 qRT‐PCR analysis

qRT‐PCR was performed as previously reported (Zhou et al., 2021). Briefly, total RNAs were extracted from tissue or cultured cells using the Trizol reagent (Invitrogen). mRNAs were reverse‐transcribed and then amplified using a real time PCR system (Applied Biosystems). The primer sequences used for real time PCR are given in Table **S1**.

### 2.11 Histology analysis

Liver and adipose tissue including BAT, inguinal white adipose tissue (iWAT) and epididymal white adipose tissue (eWAT) samples were fixed in 4% PFA after harvesting, dehydrated, and embedded in paraffin. For haematoxylin and eosin (H&E) staining and Sirius Red staining, paraffin‐embedded tissues were cut into 5 μm thick sections, de‐waxed and hydrated, then stained in haematoxylin/eosin solution.

### 2.12 Immunofluorescence

Fresh adipose tissues were isolated, post‐fixed and dehydrated with alcohol in increasing concentration: 70% for 24 h, 80% for 3 h, 90% for 4 h, 95% for 3 h and then absolute alcohol for 2 h. Adipose tissue slices with the thickness of 5 μm were cut by a microtome (Thermo Fisher Scientific), and blocked with 3% BSA, then, treated with primary antibodies TH (GeneTex, GTX634481, 1:200), YB‐1 (Cell signalling, 4202, 1:50). Subsequently, the adipose tissue slides were incubated with Alexa Fluor 488 conjugated anti‐Rabbit (Invitrogen, A21206, 1:2000) or Alexa Fluor 555 conjugated anti‐Rabbit (Invitrogen, A21428, 1:2000). All the fluorescence pictures were captured by a fluorescence microscope.

### 2.13 RNA‐seq

Total RNA was isolated and verified using NanoDrop 2000 spectrophotometer (Thermo Scientific) and Agilent 2100 Bioanalyzer (Agilent Technologies, Santa Clara, CA). Then the libraries were constructed using TruSeq Stranded mRNA LT Sample Prep Kit (Illumina, San Diego, CA) according to the manufacturer's instructions. The transcriptome sequencing and analysis were conducted by OE Biotech Co., Ltd. (Shanghai, China). Briefly, the libraries were sequenced on an Illumina Nova seq 6000 platform and 150 bp paired‐end reads were generated. Raw Reads were processed using Trimmomatic to obtain the clean reads and mapped to the mouse genome (GRCm38) using HISAT2. FPKM and read counts of each gene were obtained by Cufflinks and HTSeq‐count separately. Differential gene expression analysis was performed using the DESeq. Differentially expressed genes (DEGs) were identified based on *P* < 0.05 and fold change ≥1.3.

### 2.14 Comprehensive lab animal monitoring system

Indirect calorimetry experiments were conducted with a Comprehensive Lab Animal Monitoring System (CLAMS, Columbus Instruments). Mice in the CLAMS were fed ad libitum and maintained at either room temperature (25°C) conditions for a total of 3 days. Results of oxygen consumption, activity and RER were collected and analysed.

### 2.15 Statistical analysis

The data are expressed as mean ± SEM. Two‐tailed Student's *t* test was used to compare between two groups. When comparing the difference between multiple groups, one‐way or two‐way ANOVA was applied. Statistical differences were supposed to be significant when *P* < 0.05.

## 3 RESULTS

### 3.1 YB‐1 expression is reduced in the BAT of aged mice

**FIGURE 1:**
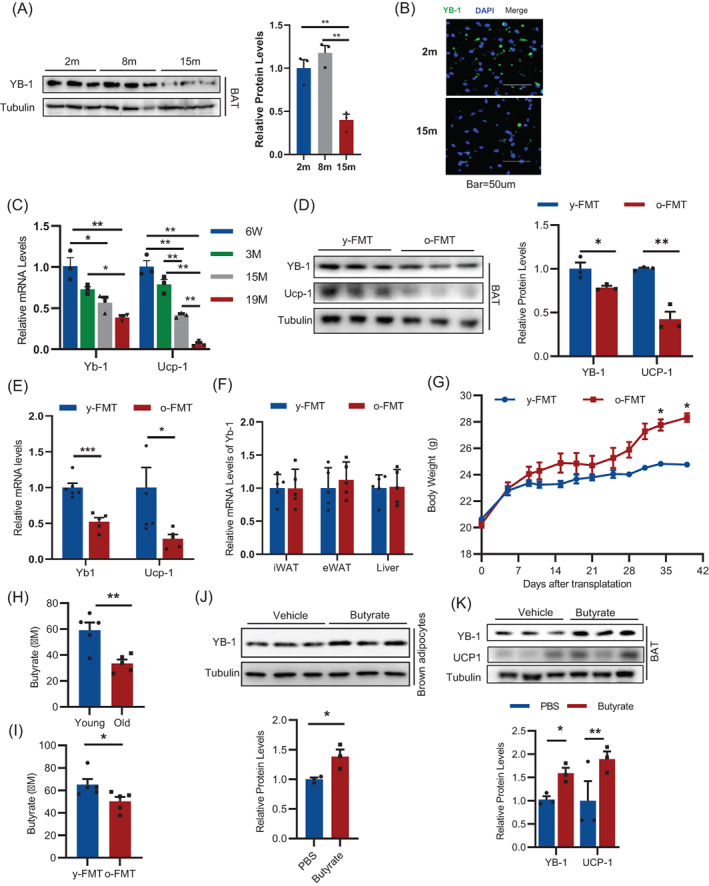
YB‐1 expression is reduced in the brown adipose tissue (BAT) of aged mice. Western blot (A) and immunofluorescence (B) analysis of YB‐1 expression in the BAT of mice during aging. (C) q‐PCR analysis of YB‐1 and UCP‐1 expression in the BAT of mice during aging. Western blot (D) and q‐PCR analysis (E) of YB‐1 and UCP‐1 expression in BAT of mice after microbiota transplantation. (F) q‐PCR analysis of YB‐1 expression in different tissues of mice after microbiota transplantation. (G) Weight change of mice after microbiota transplantation. (H) Butyrate levels in the serum of young (2‐month) and old (15‐month) mice. (I) Butyrate levels in the serum of young mice after microbiota transplantation. (J) Western blot analysis of YB‐1 expression in brown adipocyte after butyrate treatment. (K) Western blot analysis of YB‐1 and UCP‐1 expression in the BAT of mice treated with butyrate. Data are shown as the mean ± SEM. *P < 0.05, **P < 0.01, ***P < 0.001 by two‐way ANOVA or Student's *t*‐test.

To determine whether YB‐1 is related with aging‐like BAT dysfunction, we detected the expression of YB‐1 in the BAT of young and aged mice. We found that the protein and mRNA levels of YB‐1 in BAT obviously decreased with age (Figure 1A–C). Meanwhile, an age‐related reduction of UCP1 was also observed in the BAT of 15‐month‐old and 19‐month‐old mice (Figure 1C), demonstrating an age‐associated thermogenic decline. Disturbance of gut microbiota has been shown to be one of the major causes of metabolic diseases in elderly individuals. To verify whether the gut microbiota of aged mice affect the expression of YB‐1 and the function of BAT, we performed a gut microbiota transplantation experiment. Faeces from young donor (2 m) and aged donor (15 m) mice were transplanted into the young recipients (2 m), respectively. The results claimed that the expression of YB‐1 and UCP‐1 in the BAT of mice receiving faecal transplants from aged donors was lower than that received faecal transplants from young donors (Figure 1D, E). However, the expression of YB‐1 in white fat and liver is not affected (Figure 1F), indicating an organ‐specific regulation of YB‐1 by gut microbiota. In addition, we confirmed that the aged donor group had significantly higher body weight than the younger donor group (Figure 1G). These results suggest that the expression of YB‐1 in the BAT of aged individuals is possibly due to the disturbance of the gut microbiota caused by aging.

The disturbance of the gut microbiota in aging individuals leads to changes in the metabolites, some of which can enter the circulating blood and affect non‐gut tissues and organs. SCFA is one of the key intestinal flora metabolites, more than 95% of which are composed of acetate, propionate and butyrate. Among SCFAs, butyrate is a key regulator mediating BAT thermogenesis, but the specific mechanism is still uncertain.^14^, ^15^, ^16^ We thus wondered whether the reduction in butyrate account for the decreased YB‐1 expression. Therefore, we verified the effect of butyrate on YB‐1 expression in vitro and in vivo. We first determined the serum butyrate in the 2‐month‐old and 15‐month‐old mice and confirmed its decreasing in the serum (Figure 1H). In addition, the serum butyrate levels were also suppressed after of mice receiving faecal transplants from aged donors (Figure 1I). Butyrate treatment (0.5 mM) upregulated the expression of YB‐1 in brown adipocytes (Figure 1J). Mice gavaged with butyrate showed increased expression of YB‐1 in the BAT, accompanied by enhanced UCP1 expression (Figure 1K). Though other possibilities may exist, these results suggest that decrease levels of SCFAs such as butyrate in serum of aging individuals may at least in part account for the decreased YB‐1 expression in the BAT.

Together, these results suggest that YB‐1 expression in the BAT decreases during aging possibly due to the decreased butyrate acid levels.

### 3.2 Adipose‐specific YB‐1 ablation accelerates HFD‐induced obesity and insulin resistance

**FIGURE 2:**
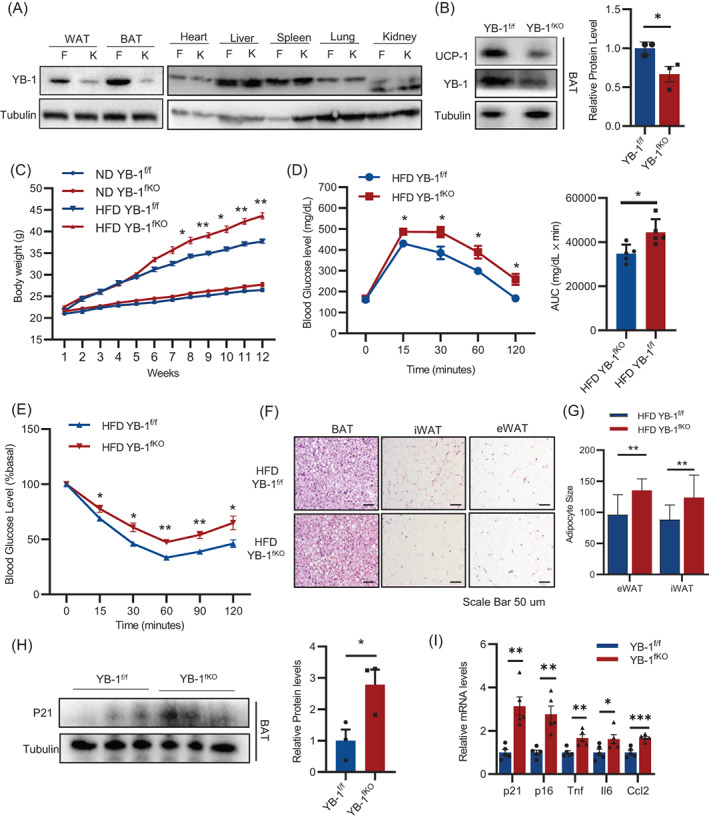
Adipose‐specific YB‐1 ablation accelerates HFD‐induced obesity and insulin resistance. (A) Western blot analysis of YB‐1 and UCP‐1 expression in different tissues of YB^f/f^ and YB^fKO^ mice. (B) YB‐1 and UCP1 expression in the brown adipose tissue (BAT) of YB^f/f^ and YB^fKO^ mice. (C) Body weight of YB^f/f^ and YB^fKO^ mice under normal chow diet (ND) or high fat diet (HFD). (D, E) GTT and ITT of YB^f/f^ and YB^fKO^ mice under HFD. (F) HE staining of BAT, iWAT and eWAT of YB^f/f^ and YB^fKO^ mice under HFD. (G) Adipocyte sizes of eWAT and iWAT of YB^f/f^ and YB^fKO^ mice under HFD. (H) Western blot analysis of p21 expression in BAT of YB^f/f^ and YB^fKO^ mice. (I) q‐PCR analysis of aging markers and SASP genes in BAT of YB^f/f^ and YB^fKO^ mice. Data are shown as the mean ± SEM. *P < 0.05, **P < 0.01, ***P < 0.001 by Student's *t*‐test.

To explore the in vivo role of adipocyte YB‐1 in BAT function, we generated fat‐specific YB‐1 knockout mice (YB‐1^fKO^) by crossing *adiponectin*‐cre mice with YB‐1 floxed mice (YB‐1^f/f^). YB‐1 was specifically suppressed in the BAT and WAT of the YB‐1^fKO^ mice, but not other tissues (Figure 2A). Of note, the expression of YB‐1 was highly enriched in the BAT compared to that in the WAT, which may indicate a more dominant role of YB‐1 in the BAT (Figure 2A). Accordingly, YB‐1^fKO^ mice displayed decreased UCP‐1 expression in the BAT (Figure 2B). To determine the effect of adipose YB‐1 on metabolic phenotypes, YB‐1^fKO^ and YB‐1^f/f^ was then subjected to normal chow diet (ND) or high‐fat diet (HFD) for 12 weeks. We observed no differences in body weight gain between the two groups under ND feeding conditions (Figure 2C). However, YB‐1^fKO^ mice gained much more body weights and fat mass when compared with the control mice after 3 months HFD feeding (Figure 2C), with impaired glucose tolerance and insulin sensitivity (Figure 2D, E). Consistently, the YB‐1^fKO^ mice displayed enlarged increased lipid accumulation in the adipose tissue and enlarged adipocyte sizes in the epididymal WAT (eWAT), inguinal WAT (iWAT) when compared with the control littermates (Figure 2F, G). To be note, YB‐1^fKO^ mice showed significantly increased expression of aging markers p16 and p21 and Senescence Associated Secretory Phenotype (SASP) genes in the BAT (Figure 2H, I).

**FIGURE 3:**
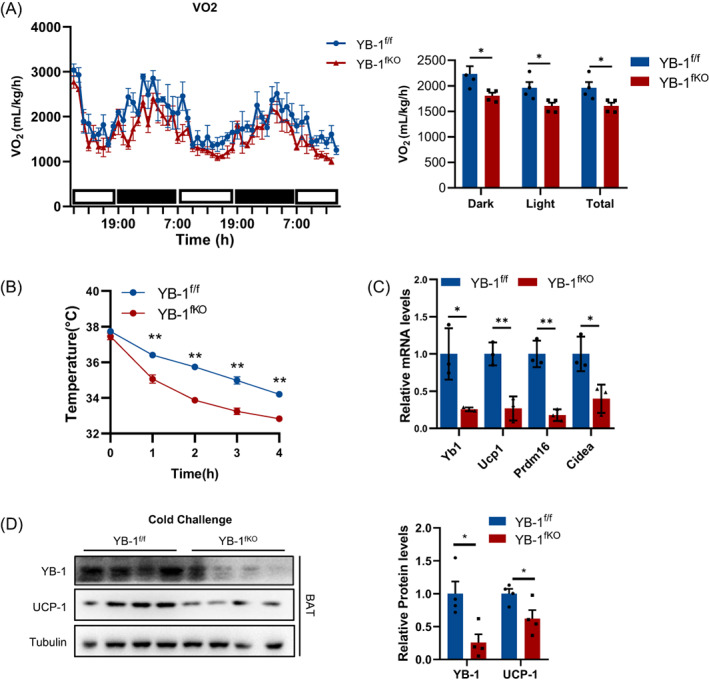
Adipose‐specific YB‐1 ablation promotes energy expenditure. (A) O_2_ consumption of YB^f/f^ and YB^fKO^ mice. (B) Core body temperature of YB^f/f^ and YB^fKO^ mice after cold challenge in 6°C. (C, D) q‐PCR and Western blot analysis of YB‐1 and thermogenesis‐related genes expression in brown adipose tissue of YB^f/f^ and YB^fKO^ mice after cold challenge in 6°C. Data are shown as the mean ± SEM. *P < 0.05, **P < 0.01, ***P < 0.001 by covariance analysis (A) or Student's *t*‐test.

Obesity was caused by the imbalance between energy intake and energy output. We further wondered how YB‐1 deficiency greatly promoted obesity. Since YB‐1 deficiency showed no effect on food intake or activity (Figure S1B, C), we therefore infer that YB‐1 deficiency may suppress energy expenditure. To address this concern, metabolic cages experiments were performed on YB‐1^fKO^ and YB‐1^f/f^ control littermates fed with HFD for 5 weeks, when mice were about to show body weight differences. Results suggested that YB‐1^fKO^ showed significantly decreased oxygen consumption when compared with their YB‐1^f/f^ control littermates (Figure 3A, B). Moreover, we exposed YB‐1^fKO^ and YB‐1^f/f^ to 6°C for 24 h. YB‐1^fKO^ mice showed a worsened core body temperature decreasing rate and impaired cold tolerance (Figure 3B). Consistently, cold stimulation robustly enhanced mRNA and protein levels of thermogenesis‐related genes in the BAT of control mice while this promotion was significantly ablated in YB‐1^fKO^ mice (Figure 3C, D). These results suggest that YB‐1 deficiency in adipocytes leads to disrupted energy metabolism and accelerates diet‐induced obesity.

### 3.3 Overexpression of YB‐1 in BAT abolished obesity‐induced BAT dysfunction in aged mice

**FIGURE 4:**
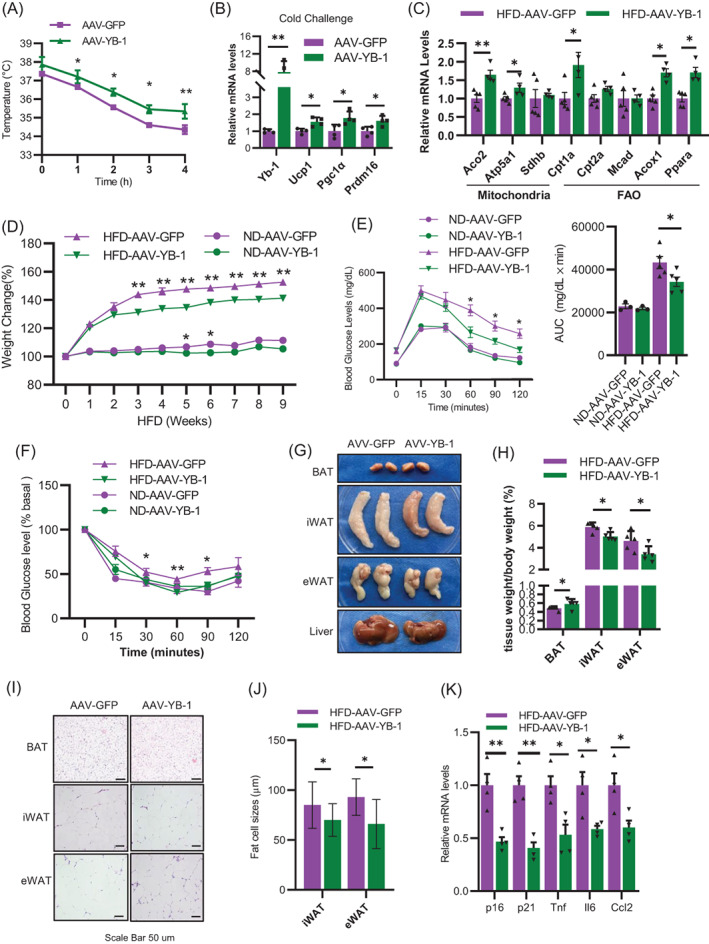
Overexpression of YB‐1 in brown adipose tissue (BAT) abolished obesity‐induced BAT dysfunction in aged mice. (A) Core body temperature of control mice and AAV‐YB‐1 mice after cold challenge in 6°C. (B) q‐PCR analysis of YB‐1 and thermogenesis‐related genes expression in BAT of control mice and AAV‐YB‐1 mice after cold challenge in 6°C. (C) q‐PCR analysis of mitochondrial functions and fatty acid oxidation related genes expression in BAT of control mice and AAV‐YB‐1 mice. (D) Body weight of control mice and AAV‐YB‐1 mice under normal chow diet (ND) or high fat diet (HFD). (E, F) GTT and ITT of control mice and AAV‐YB‐1 mice under ND and HFD. (G) Pictures of BAT, iWAT, eWAT and liver from control mice and AAV‐YB‐1 mice under HFD. (H) BAT, iWAT, eWAT and liver to body mass ratio of control mice and AAV‐YB‐1 mice under HFD. (I) HE staining of BAT, iWAT and eWAT of control mice and AAV‐YB‐1 mice under HFD. (J) Adipocyte sizes of eWAT and iWAT of control mice and AAV‐YB‐1 mice under HFD. (K) q‐PCR analysis of p16 and p21 expression in BAT control mice and AAV‐YB‐1 mice under HFD. (K) q‐PCR analysis of aging markers and SASP genes in the BAT of control mice and AAV‐YB‐1 mice under HFD. Data are shown as the mean ± SEM. *P < 0.05, **P < 0.01, ***P < 0.001 by two‐way ANOVA or Student's *t*‐test.

In order to clarify whether YB‐1 counteract BAT aging and metabolic dysfunction, we overexpressed YB‐1 in the BAT of aged mice by fat‐pad injection of AAV‐FABP4‐YB‐1 (AAV‐YB‐1), or AAV‐ FABP4‐GFP in the control group (AAV‐GFP), which allowing for adipocyte‐specific overexpression of YB‐1 in the BAT. Compared with control mice, AAV‐YB‐1 mice showed improved cold tolerance and enhanced thermogenic gene expression (Figure 4A, B). Mitochondrial functions and fatty acid oxidation (FAO) related genes in BAT, such as ACO2, Atp5a1, Cpt1a, ACOX1, and PPARA were also found to be elevated after overexpression of YB‐1 (Figure 4C), indicating improved brown adipocyte thermogenic function.

To further demonstrate a protective role of YB‐1 in metabolic benefits, mice injected with AAV‐GFP or AAV‐YB‐1 were subjected to ND or HFD feeding. We observed that in the early stage of HFD feeding (3 weeks), the body weight of AAV‐YB‐1‐treated mice was already significantly higher than AAV‐GFP‐treated mice (Figure 4D). The GTT and ITT experiments performed after 9 weeks of HFD indicated that AAV‐YB‐1 mice had higher glucose tolerance and insulin sensitivity (Figure 4E, F). Consistently, the volume and tissue weight ratio of iWAT and eWAT in AAV‐YB‐1 mice were much smaller and decreased when compared to that of AAV‐GFP mice (Figure 3G, H). HE staining of paraffin sections of adipose tissue showed smaller lipid droplets in iWAT and eWAT in AAV‐YB‐1 group (Figure 4I, J). Food intake was recorded and found that there was no difference in food intake between the two groups of mice (Figure S2A, B), indicating that the difference in obesity was not due to food intake. Although subtle body weight differences were observed in chow‐fed mice (Figure 4D), no metabolic phenotypic changes were observed (Figure 4E, F). The reduction of senescence‐related genes in the AAV‐YB‐1 group also suggested that overexpression of YB‐1 could slow down BAT senescence (Figure 4K). Together，this demonstrates that overexpression of YB‐1 in aged mice not only improves BAT function, but also improves systemic metabolic disorders.

### 3.4 YB‐1 deficiency decreased sympathetic innervation in the BAT

**FIGURE 5:**
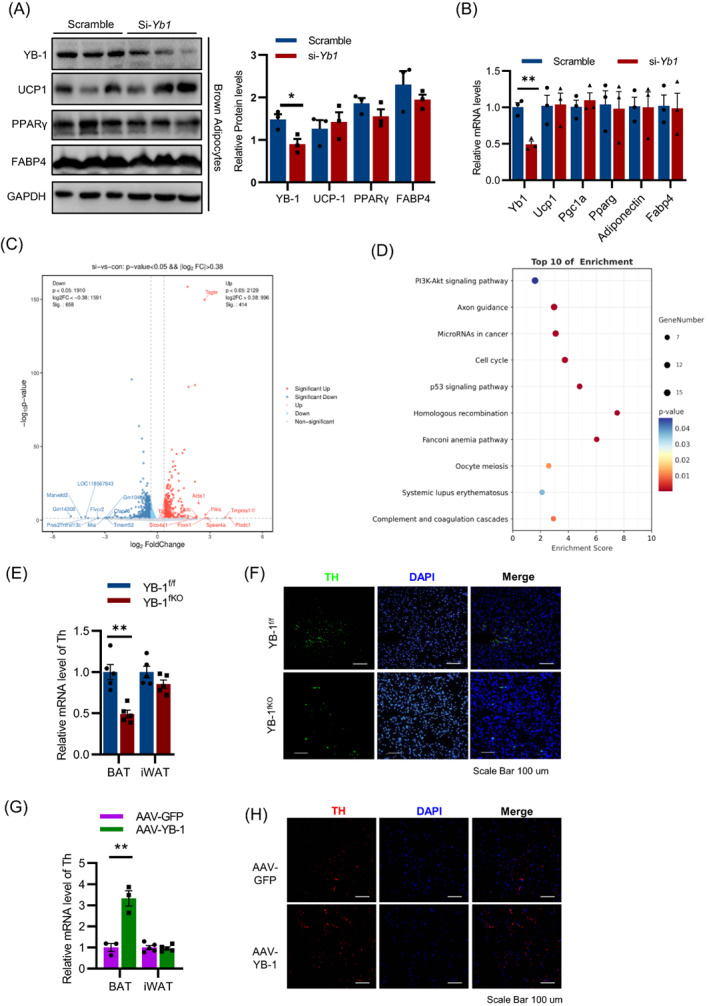
YB‐1 deficiency had no direct effect on UCP1 expression but decreased axon guidance‐related genes. Western blot (A) and q‐PCR (B) analysis of adipogenic related genes expression in differentiated brown adipocyte transfected with YB‐1 siRNA or scramble control. (C) Volcanic map of up‐regulated and down‐regulated genes in YB‐1 deficiency brown adipocytes compared with the control group. Differentially expressed genes showed are with at least 1.3 fold changes and *P* < 0.05. (D) Top 10 pathways from KEGG pathway enrichment analysis. (E) q‐PCR analysis of TH expression in brown adipose tissue (BAT) and iWAT of YB^f/f^ and YB^fKO^ mice. (F) Immunofluorescence analysis of TH expression in BAT of YB^f/f^ and YB^fKO^ mice. (G) q‐PCR analysis of TH expression in BAT and iWAT of AAV‐GFP mice and AAV‐YB‐1 mice. (H) Immunofluorescence analysis of TH expression in BAT of AAV‐GFP mice and AAV‐YB‐1 mice. Data are shown as the mean ± SEM. *P < 0.05, **P < 0.01, ***P < 0.001 by Student's *t*‐test.

We next investigate the mechanism of YB‐1 in restoring BAT function. Consistent with previous reports, YB‐1 deficient in SVF abolished brown adipogenesis and UCP1 expression (Figure S3A, B). Surprisingly, siRNA‐mediated knockdown of YB‐1 in mature brown adipocytes showed similar UCP1 expression and adipogenic function compared with control adipocytes (Figure 5A, B). Given YB‐1^fKO^ displayed impaired cold sensitivity, these discrepancies led us hypothesized that YB‐1 in brown adipocytes may regulate BAT thermogenic function via communication with other cell types. YB‐1 is reported as a critical RNA binding protein which regulates gene expression via alternative splicing or maintaining the stability of target mRNAs. We thus performed RNA‐seq of control and YB‐1‐deficient brown adipocytes to profile the DEGs. A total of 1072 genes with At least a 1.3‐fold change were identified, among which 658 and 414 genes were found to be up‐regulated and down‐regulated, respectively, in the si‐Yb‐1 group compared with the control group (Figure 5C). Go analysis of the overlapping genes showed that Top3 enriched pathways were PI3K‐AKT signalling pathway, axon guidance, microRNAs in cancer (Figure 5D). Among them, we paid our attention to axon guidance, a pathway related closely related with sympathetic innervation and energy metabolism.

Tyrosine hydroxylase (TH), the rate‐limiting enzyme in catecholamine biosynthesis, is a marker for sympathetic innervation Consistent with the RNA‐seq results, we found the BAT from YB‐1^fKO^ mice showed ablated TH expression, whereas this ablation was quite mild in iWAT (Figure 5E, F), possibly due to the lower YB‐1 expression and sympathetic innervation in iWAT compared with BAT. By contrast, mRNA levels of TH in BAT were enhanced in AAV‐YB‐1 mice compared with control mice, whereas iWAT showed no changes (Figure 5G, H). Together, these data suggest that YB‐1 in mature brown adipocytes regulate thermogenesis mainly via promoting sympathetic innervation.

### 3.5 YB‐1 regulates sympathetic innervation through promoting Slit2 expression

**FIGURE 6:**
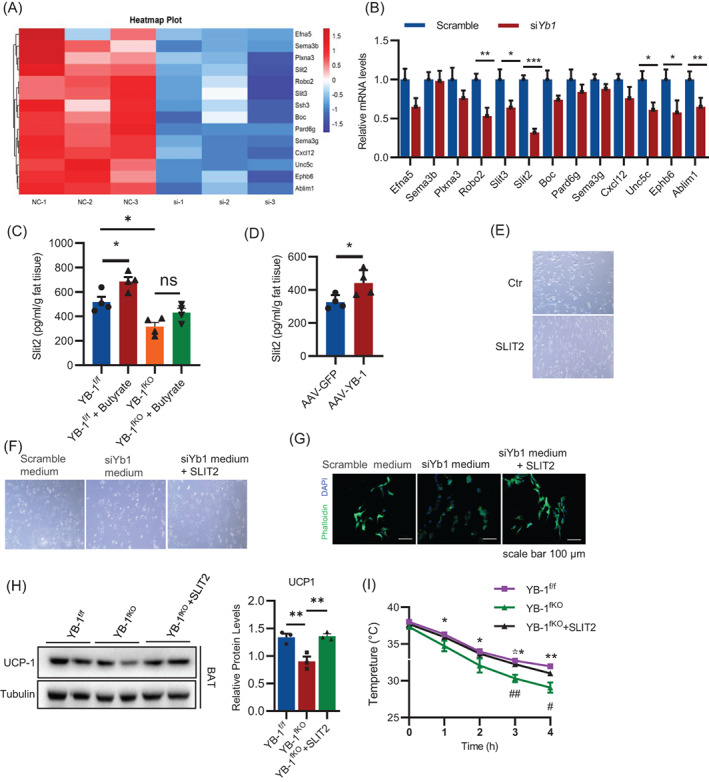
Slit2 produced by brown adipocytes is essential for YB‐1 mediated sympathetic innervation. (A) Heatmap of differentially expressed genes in the axon guidance pathway. (B) q‐PCR analysis of axon guidance‐related genes expression in control brown adipocyte and YB‐1 deficiency adipocyte. (C) ELISA analysis of Slit2 expression in supernatant of control brown adipocyte and YB‐1 deficiency brown adipocyte treated with or without butyrate. (D) ELISA analysis of Slit2 expression in supernatant of control brown adipocyte and YB‐1‐overexpressed brown adipocyte. (E) Pictures of differentiated PC12 cell treated with recombinant Slit2 proteins. (F) Pictures of undifferentiated PC12 cells treated with scramble medium, siYb1 medium and siYb1 medium plus Slit2 protein. (G) Phalloidin staining of microfilaments that indicate neurite outgrow in PC12 cells treated with scramble medium, si‐*Yb1* medium and si‐*Yb1* medium plus Slit2 protein. Scale bar: 100 μm. (H) Western blot analysis of UCP‐1 expression in brown adipose tissue of YB^f/f^, YB^fKO^, mice and YB^fKO^ mice treated with Slit2. (I) Core body temperature of YB^f/f^, YB^fKO^ mice and YB^fKO^ mice treated with Slit2. Data are shown as the mean ± SEM. *P < 0.05, **P < 0.01, ***P < 0.001 by two‐way ANOVA or Student's *t*‐test.

We next try to depict the exact mechanism that responsible for the sympathetic innervation regulated by YB‐1. The decreased expression of genes involved in axon guidance including *Efna5, Sema3b, Plxna3, Slit2, Robo2, Slit3* (Figure 6A). qPCR analysis was performed to validate the expression of these gene (Figure 6B).

Among the candidates in axon guidance pathways identified by RNA‐seq, *Slit2*, a secreted factor reported to promote angiogenesis and neuron,^17^ was the mostly significantly decreased by YB‐1 deficiency and were chosen for further study (Figure 6B). ELISA analysis demonstrated that Slit2 expression was significantly reduced in the supernatant of YB‐1‐knockout brown adipocytes compared to that of control adipocytes (Figure 6C). Moreover, butyrate treatment magnified Slit2 expression in brown adipocytes while this effect was abolished in YB‐1 knockout adipocytes, demonstrating that YB‐1 was critical for mediating the effects of butyrate (Figure 6C). Moreover, overexpression of YB‐1 in adipocytes promoted Slit2 expression (Figure 6D).

To confirm the function of Silt2 on neurogenesis, we introduced PC12 cells, a rat adrenal chromaffin cell that can be induced to differentiate into neurons.^18^ Differentiated PC12 cells can synthesize and secrete catecholamine neurotransmitters, and are often used to study the function of sympathetic nerves.^19^ We added Slit2 proteins to PC12 cell culture medium and found that Slit2 could induce the differentiation and neurite outgrowth of PC12 cells (Figure 6E). Next, we cultured PC12 cells with supernatants collected from adipocytes transfected with scramble or si‐YB‐1. Cell morphology and phalloidin staining of microfilaments suggested that compared with control supernatant, supernatant collected from YB‐1 knockout adipocytes inhibited neural differentiation, but the addition of Slit2 rescued this situation (Figure 6F, G). Next, we injected Slit2 protein into YB‐1^fko^ mice intraperitoneally three times a week for 2 weeks and found that Slit2 restored the reduction of UCP1 expression in the BAT caused by YB‐1 knockout (Figure 6H). Moreover, the cold acclimation was improved in YB‐1^fKO^ mice treated with Slit2 (Figure 6I). Together, the above results showed that YB‐1 regulates sympathetic innervation by promoting Slit2 expression.

### 3.6 Sciadopitysin maintains YB‐1 expression and ameliorate age‐related metabolic dysfunction

**FIGURE 7:**
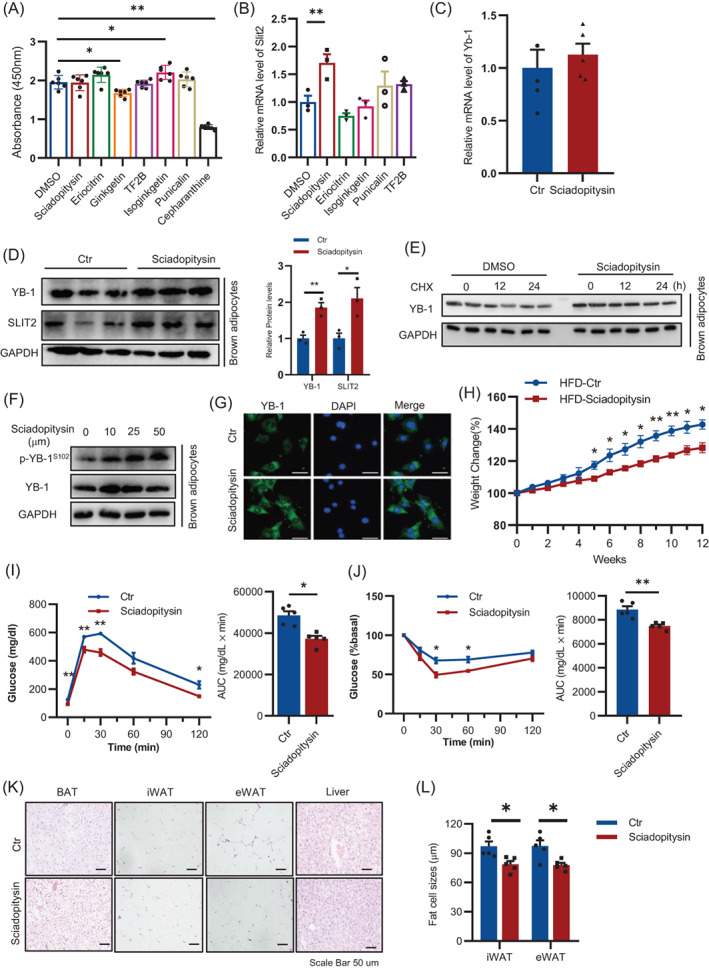
A natural compound, sciadopitysin, maintains YB‐1 expression and ameliorates age‐related metabolic dysfunction. (A) CCK8 assay of adipocytes treated with different natural small molecular compounds. (B) q‐PCR analysis of Slit2 expression in adipocytes treated with different natural small molecular compounds. (C) q‐PCR analysis of Yb‐1 expression in brown adipocyte treated with sciadopitysin. (D) Western blot analysis of YB‐1 and Slit2 expression in brown adipocyte treated with sciadopitysin. (E) Western blot analysis of YB‐1 expression in brown adipocyte treated with sciadopitysin and CHX. (F) Western blot analysis of YB‐1 and phosphorylated YB‐1 in brown adipocyte treated with sciadopitysin. (G) Immunofluorescence analysis of YB‐1 expression in brown adipocyte treated with sciadopitysin. (H) Body weight of control mice and sciadopitysin treated mice under high fat diet (HFD) feeding conditions. (I, J) GTT and ITT of control mice and sciadopitysin treated mice after HFD feeding for 12 weeks. (K) HE staining of brown adipose tissue, iWAT, eWAT and liver of control mice and sciadopitysin treated mice after HFD feeding for 12 weeks. (L) Adipocyte sizes of eWAT and iWAT of control mice and sciadopitysin treated mice after HFD feeding for 12 weeks. Data are shown as the mean ± SEM. *P < 0.05, **P < 0.01, ***P < 0.001 by two‐way ANOVA or Student's *t*‐test.

To search the potential therapeutic strategy that alleviating age‐related BAT dysfunction by targeting YB‐1, we performed molecular docking to screen the natural small molecular compounds that interacted with mouse YB‐1 as described.^11^ We chose seven top‐ranked small molecules that are related to anti‐oxidation, anti‐inflammation and anti‐aging (Figure 7A). Among these candidates, five compounds including sciadopitysin, eriocitrin, theaflavin‐3‐gallate, isoginkgetin and punicalin showed no adverse effect on adipicyte viability by CCK8 assay (Figure 7A). But only sciadopitysin showed enhanced expression of Slit2 (Figure 7B). Thus, we chose sciadopitysin for further study. The protein, but not mRNA levels YB‐1, was also enhanced by Sciadopitysin treatment in brown adipocytes (Figure 7C, D). To investigate the mechanism by which sciadopitysin treatment increased the protein level of YB‐1, we blocked the protein synthesis in brown adipocytes by cycloheximide (CHX) and found that sciadopitysin treatment slow down the degradation of YB‐1 (Figure 7E). Additionally, phosphorylation of YB‐1 at the serine 102 residue, which is essential for YB‐1 nuclear translocation and transcriptional activation of target genes, was also enhanced following sciadopitysin treatment (Figure 7F, G). To further investigate whether sciadopitysin administration could alleviate age‐related BAT dysfunction, 12‐month‐old C57/BL6J mice were administrated with sciadopitysin at 10 mg/kg/day or with vehicle. Under ND feeding conditions, sciadopitysin treatment for 4 weeks showed a decreasing trend on body weight (Figure S4A). However, sciadopitysin treatment suppressed p16 and p21 expression in BAT, while enhanced BAT sympathetic innervation and thermogenic gene expression (Figure S4B, C). Importantly, after HFD feeding for 12 weeks, mice treated with sciadopitysin showed much less weight gain and correspondingly improved metabolic phenotypes including improved glucose tolerance, insulin sensitivity, eWAT and liver mass, and adipocyte sizes (Figure 7H–L). These results demonstrate that by targeting YB‐1 expression and nucleus translocation, sciadopitysin ameliorates BAT aging, enhanced sympathetic innervation and thermogenesis, leading to metabolic homeostasis.

## 4 DISCUSSION

**FIGURE 8:**
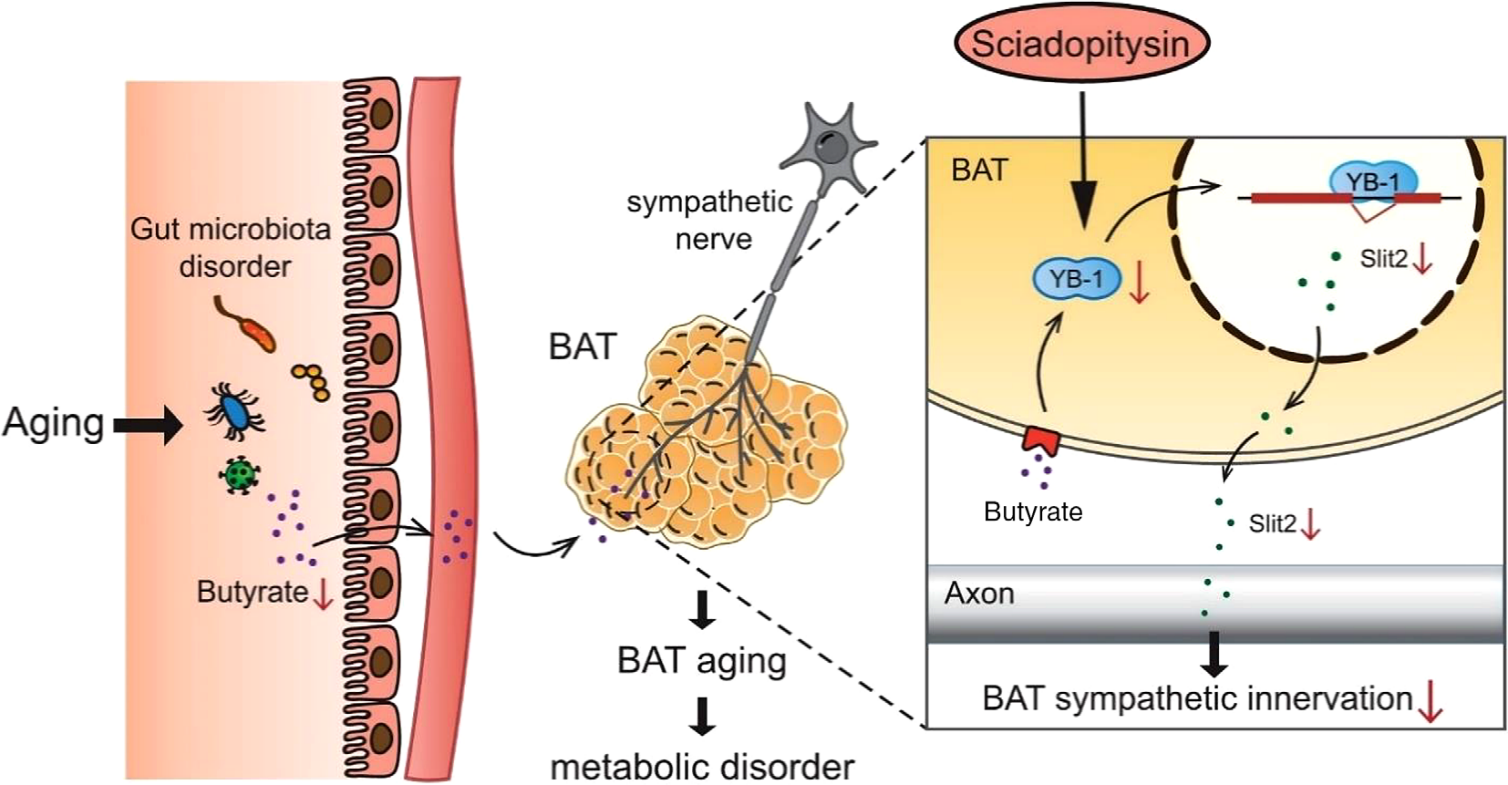
Proposed mechanism of YB‐1 linking aging‐related gut microbiota dysfunction and sympathetic denervation. YB‐1 expression in the brown adipose tissue (BAT) gradually decreases during aging due to the low contents of gut microbiota metabolite butyrate. YB‐1 magnifies Slit2 expression to promote BAT thermogenic function via potentiating sympathetic innervation. A small molecular compound Sciadopitysin maintains YB‐1 expression and is beneficial to alleviate the aging‐related BAT dysfunction and metabolic disorders.

Identifying the key regulatory factors in the occurrence and development of adipose tissue senescence and determining whether the key regulatory factors can improve metabolic disorders will provide a new approach for the pathogenesis and prevention of age‐related metabolic disorders. In this study, we found that decreased serum butyrate levels in aging mice resulted in decreased YB‐1 expression in the BAT, which impairs BAT function by inhibiting sympathetic innervation and aggravates aging‐related metabolic syndrome (Figure 8). Importantly, we have identified a natural compound, JS, could maintain the expression of YB‐1 in the BAT, thus alleviating aging‐related BAT dysfunction (Figure 8).

The sympathetic nervous system innervates BAT to regulate their function and maintain homeostasis, whereas neighbouring cells in BAT also produce neurotrophic factors to promote sympathetic innervation.^1^, ^2^ A fat‐derived “adipokine” neurotrophic factor neurotrophic 3 (NT‐3) and M2 macrophages‐derived Slit3 were found to regulate sympathetic neuron growth and innervation in adipose tissue, respectively.^20^, ^21^ Of note, we found Slit2 as a downstream target of YB‐1 in BAT while there was only moderate decrease in Slit3 expression. Slit3 can bind to its receptor ROBO, which is also a receptor for Slit2, to regulate and guide the distribution of nerve axons.^22^ Thought we cannot rule out the possibility that Slit3 may also involve in the function of YB‐1, we believe that Slit2 produced by brown adipocytes at least partially mediate the BAT sympathetic innervation by YB‐1.

YB‐1 regulates the expression of senescence‐related genes p16Ink4a, p21Cip1 and p53, and YB‐1 knockout leads to severe embryonic premature aging and a lethal phenotype.^23^ Consistent with previous reports,^12^, ^13^, ^24^ we have found YB‐1 knockdown in brown preadipocytes significantly impaired brown adipogenesis and thermogenic potential, however, such effects were not observed when YB‐1 was knockout in mature adipocytes. Given the YB‐1^fKO^ mice, which specifically depleted YB‐1 in mature adipocytes, showed decreased thermogenic capacity and cold tolerance, it is thus interesting to explore the external mechanisms of mature adipocyte YB‐1 deficiency in thermogenic function. Using RNA‐sequencing, we have defined axon guidance as a critical pathway that intrinsically affected by YB‐1 deficiency in mature adipocytes. Several genes were found impaired expression with the repression of YB‐1, including Silt2. In vitro coculture experiments further verified that YB‐1 deficiency suppressed TH expression. However, silt2 could rescues the neurogenesis deficits caused by YB‐1 deficiency. Our study found that YB‐1 in BAT does not affect the brown adipocytes themselves, but indirectly promotes the expression of neurogenesis‐related protein Slit2 which affecting the axon guidance of sympathetic nerves, and then enhances the function of BAT.

To screen naturally occurring small‐molecule compounds that target and stabilize YB‐1, we have ever performed molecular docking and virtual screening between YB‐1 and the natural small molecular compounds library of Target Molecule (Target Mol) that contains more than 6000 compounds. We have ever identified theaflavin‐3‐gallate that could enter into the pocket‐like structure of YB‐1 to maintain YB‐1 expression. Sciadopitysin, an extract of Ginkgo biloba that has anti‐inflammatory and anti‐tumour effects, was another small molecule identified through that molecular docking. In this study, we found Sciadopitysin, but not theaflavin‐3‐gallate, maintains YB‐1 expression and promoted Slit2 expression. We found that Sciadopitysin administered by intraperitoneal injection in aged mice could alleviate metabolic syndrome such as aging and obesity in mice, thus presenting a potential treatment for BAT aging and age‐related metabolic homeostasis.

Taken together, this study reveals that the YB‐1, which is suppressed by gut microbiota, controls BAT aging and metabolic homeostasis via promoting BAT sympathetic innervation. Targeting YB‐1 may offer a novel treatment strategy for age‐related metabolic disorders.

## AUTHOR CONTRIBUTIONS

Conceptualization: Haiyan Zhou and Yuan Xiao; Formal analysis: Yan Huang and Ruoyu Zhou; Funding acquisition: Haiyan Zhou; Investigation: Ruoyu Zhou and Xu Feng; Methodology: Ruoyu Zhou and Yan Huang; Supervision: Haiyan Zhou and Yuan Xiao; Validation: Liwen Wang and Rui Zhou; Roles/writing–original draft: Ruoyu Zhou and Yan Huang; Writing–review & editing: Haiyan Zhou, Yuan Xiao, and Genqing Xie.

## CONFLICT OF INTEREST STATEMENT

The authors declare no conflicts of interest.

## Supporting information


**Figure S1.** YB‐1 deficiency in adipocytes did not affect food intake or activity. (A) Food intake of YB^f/f^ and YB^fKO^ mice after HFD feeding for 5 weeks. (B) Activity of YB^f/f^ and YB^fKO^ mice after HFD feeding for 5 weeks.
Data are shown as the mean ± SEM. *P < 0.05, **P < 0.01, ***P < 0.001 by covariance analysis (C) or Student's *t*‐test.
**Figure S2.** YB‐1 overexpression in the BAT did not affect food intake. (A, B) Food intake of control mice and YB‐1 overexpression mice under ND and HFD. Data are shown as the mean ± SEM. *P < 0.05, **P < 0.01, ***P < 0.001 by Student's *t*‐test.
**Figure S3.** YB‐1 deficiency in pre‐adipocytes abolishes adipogenesis and thermogenic function. (A) Western blot analysis of adipogenic and thermogenic related genes expression in adipocytes differentiated from SVF transfected with shYB‐1 adenovirus or scramble adenovirus. (B) Oil‐red staining of adipocytes differentiated from SVF transfected with shYB‐1 adenovirus or scramble adenovirus.
**Figure S4.** Sciadopitysin treatment suppressed BAT aging but promoted thermogenic gene expression under normal chow diet feeding conditions. (A) Body weight of control mice and sciadopitysin treated mice under ND feeding conditions. (B, C) q‐PCR analysis of *p16*, *p21*, *Th, Ucp‐1*, and *Pgc1a* expression in BAT of mice control mice and sciadopitysin treated mice under ND feeding conditions. Data are shown as the mean ± SEM. *P < 0.05, **P < 0.01, ***P < 0.001 by Student's *t*‐test.

## Data Availability

The RNA‐Seq data and RIP‐Seq data produced in this paper have been deposited in the Sequence Read Archive database and will be available upon acceptance.
