# Supplementary material for: Decreased YB‐1 expression denervates brown adipose tissue and contributes to age‐related metabolic dysfunction
Source: Cell Prolif. 2023 Jun 15;57(1):e13520. doi: 10.1111/cpr.13520 (PMC10771110; doi:10.1111/cpr.13520)
Supplement: Supplementary file 1 — Figure S1. YB‐1 deficiency in adipocytes did not affect food intake or activity. (A) Food intake of YBf/f and YBfKO mice after HFD feeding for 5 weeks. (B) Activity of YBf/f and YBfKO mice after HFD feeding for 5 weeks. Data are shown as the mean ± SEM. *P < 0.05, **P < 0.01, ***P < 0.001 by covariance analysis (C) or Student's t‐test. Figure S2. YB‐1 overexpression in the BAT did not affect food intake. (A, B) Food intake of control mice and YB‐1 overexpression mice under ND and HFD. Data are shown as the mean ± SEM. *P < 0.05, **P < 0.01, ***P < 0.001 by Student's t‐test. Figure S3. YB‐1 deficiency in pre‐adipocytes abolishes adipogenesis and thermogenic function. (A) Western blot analysis of adipogenic and thermogenic related genes expression in adipocytes differentiated from SVF transfected with shYB‐1 adenovirus or scramble adenovirus. (B) Oil‐red staining of adipocytes differentiated from SVF transfected with shYB‐1 adenovirus or scramble adenovirus. Figure S4. Sciadopitysin treatment suppressed BAT aging but promoted thermogenic gene expression under normal chow diet feeding conditions. (A) Body weight of control mice and sciadopitysin treated mice under ND feeding conditions. (B, C) q‐PCR analysis of p16, p21, Th, Ucp‐1, and Pgc1a expression in BAT of mice control mice and sciadopitysin treated mice under ND feeding conditions. Data are shown as the mean ± SEM. *P < 0.05, **P < 0.01, ***P < 0.001 by Student's t‐test. [file CPR-57-e13520-s001.pdf]

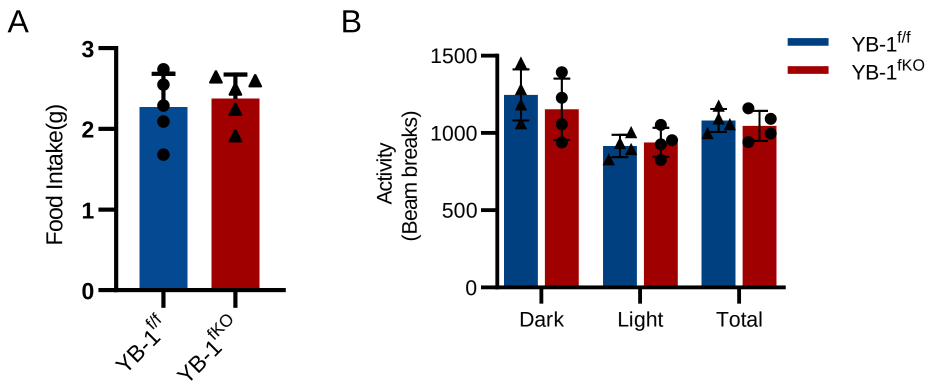

Supplementary Figure 1 YB-1 deficiency in adipocytes did not affect food intake or activity

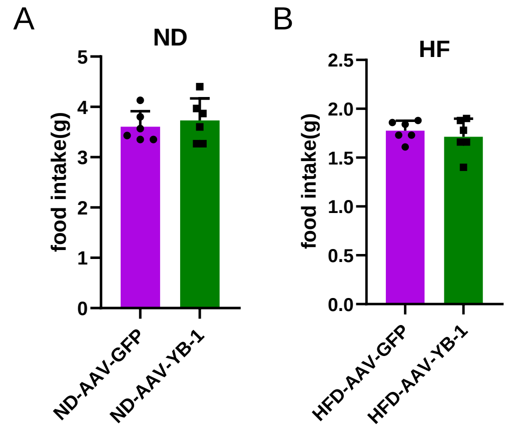

Supplementary Figure 2 YB-1 overexpression in the BAT did not affect food intake.

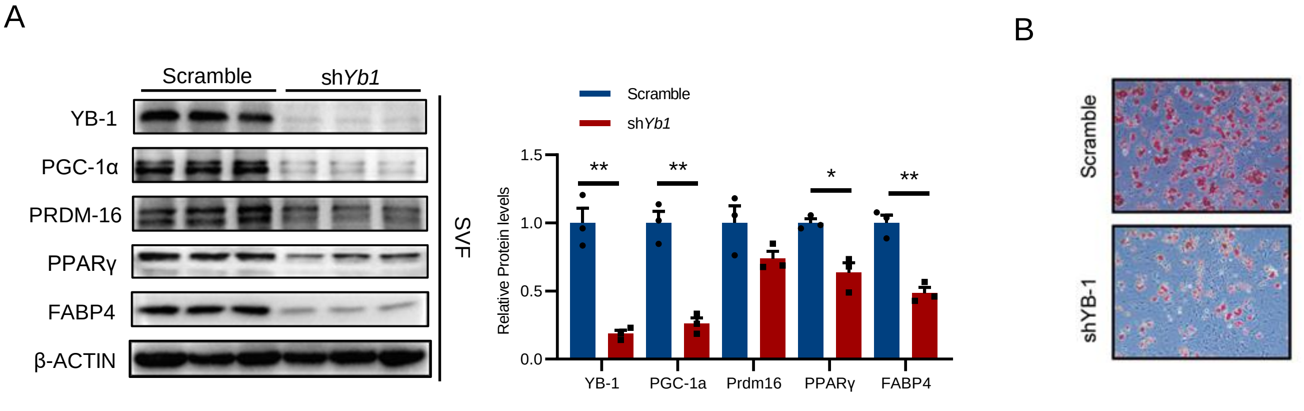

Supplementary Figure 3 YB-1 deficiency in pre-adipocytes abolishes adipogenesis and thermogenic function.

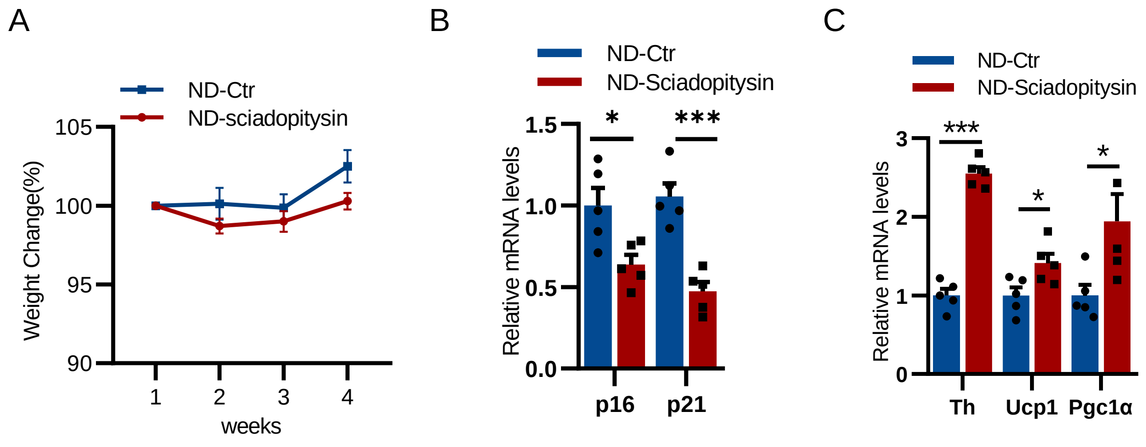

Supplementary Figure 4 Sciadopitysin treatment suppressed BAT aging but promoted thermogenic gene expression under normal chow diet feeding conditions.

Supplementary Table 1 Primer sequences used for real-time PCR

Gene	Primers	Sequences
Mouse β-actin	Forward primer	GATCATTGCTCCTCCTGAGC
	Reverse primer	ACTCCTGCTTGCTGATCCAC
Mouse Yb-1	Forward primer	CAGACCGTAACCATTATAGACGC
	Reverse primer	ATCCCTCGTTCTTTTCCCCAC
Mouse Pgc1a	Forward primer	TATGGAGTGACATAGAGTGTGCT
	Reverse primer	CCACTTCAATCCACCCAGAAAG
Mouse Ucp1	Forward primer	AGGCTTCCAGTACCATTAGGT
	Reverse primer	CTGAGTGAGGCAAAGCTGATTT
Mouse p21	Forward primer	GCAGATCCACAGCGATATCC
	Reverse primer	CAACTGCTCACTGTCCACGG
Mouse p16	Forward primer	TTGCCCATCATCATCACCT
	Reverse primer	GGGTTTTCTTGGTGAAGTTCG
Mouse Prdm16	Forward primer	CCACCAGCGAGGACTTCAC
	Reverse primer	GGAGGACTCTCGTAGCTCGAA
Mouse Cidea	Forward primer	TGACATTCATGGGATTGCAGAC
	Reverse primer	GGCCAGTTGTGATGACTAAGAC
Mouse Th	Forward primer	CCAAGGTTCATTGGACGGC
	Reverse primer	CTCTCCTCGAATACCACAGCC
Mouse Aco2	Forward primer	GCCCAGATGGCTATGCTACAG
	Reverse primer	CGCAGGTCTTTCTCACCCC
Mouse Atp5a1	Forward primer	TCTCCATGCCTCTAACACTCG
	Reverse primer	CCAGGTCAACAGACGTGTCAG
Mouse Sdhb	Forward primer	CTGAATAAGTGCGGACCTATGG
	Reverse primer	AGTATTGCCTCCGTTGATGTTC
Mouse Cpt1a	Forward primer	AGATCAATCGGACCCTAGACAC
	Reverse primer	CAGCGAGTAGCGCATAGTCA
Mouse Cpt2a	Forward primer	CAGCACAGCATCGTACCCA
	Reverse primer	TCCCAATGCCGTTCTCAAAAT
Mouse Mcad	Forward primer	ATGCCTGTGATTCTTGCTGGA
	Reverse primer	ACATCTTCTGGCCGTTGATAAC

Mouse Acox1	Forward primer	CCGCCACCTTCAATCCAGAG
	Reverse primer	CAAGTTCTCGATTTCTCGACGG
Mouse Ppara	Forward primer	TACTGCCGTTTTCACAAGTGC
	Reverse primer	AGGTCGTGTTCACAGGTAAGA
Mouse Fabp4	Forward primer	AAGGTGAAGAGCATCATAACCCT
	Reverse primer	TCACGCCTTTCATAACACATTCC
Mouse Pparg	Forward primer	TCGCTGATGCACTGCCTATG
	Reverse primer	GAGAGGTCCACAGAGCTGATT
Mouse Efna5	Forward primer	ACACGTCCAAAGGGTTCAAGA
	Reverse primer	GTACGGTGTCATTTGTTGGTCT
Mouse Sema3b	Forward primer	GTAGCAGGGCTAGGGGATACT
	Reverse primer	AAGGCTTCATAACAGCAGGTC
Mouse Plxna3	Forward primer	CAGATACCACTCTGACTCACCT
	Reverse primer	GGCCCGTAGCTCAGTTAGG
Mouse Robo2	Forward primer	TGATGGATCTCGTCTTCGTCA
	Reverse primer	GTCGGCCCTCTGCTTTACAG
Mouse Slit3	Forward primer	TGCCCCACCAAGTGTACCT
	Reverse primer	CGCCTCTCTCGATGATGCT
Mouse Slit2	Forward primer	AGCACCATCGAGAGGGGAG
	Reverse primer	GATCAAGCCGGTAGAGCTTCG
Mouse Boc	Forward primer	AGAGAGCGGCCTATACTTACAC
	Reverse primer	AAGCGCCAAGTCACGTTCA
Mouse Pard6g	Forward primer	CATAAGTCTCAGACCCTACGCT
	Reverse primer	GGTCACCTCGGTGTTAGAGATG
Mouse Sema3g	Forward primer	GGTTCCCTAGACCTCCAAGTC
	Reverse primer	GTCTTTTCCCTTGCGGACACA
Mouse Cxcl12	Forward primer	TGCATCAGTGACGGTAAACCA
	Reverse primer	TTCTTCAGCCGTGCAACAATC
Mouse Unc5c	Forward primer	CTGCGGACTGGGACTAGGATA
	Reverse primer	GGTTTCTGGGAGTTCGTGAAAA
Mouse Ephb6	Forward primer	GCTCCTGGTTCTAGGGTCATC
	Reverse primer	CCAGCCAATCTCAGAGGTCT
Mouse Ablim1	Forward primer	AGATGTCCGGGATCGGATGAT
	Reverse primer	GACGGCTCATTGCCAGGTC
